# Influence of Plant Extract Addition to Marinades on Polycyclic Aromatic Hydrocarbon Formation in Grilled Pork Meat

**DOI:** 10.3390/molecules27010175

**Published:** 2021-12-28

**Authors:** Anna Onopiuk, Klaudia Kołodziejczak, Monika Marcinkowska-Lesiak, Iwona Wojtasik-Kalinowska, Arkadiusz Szpicer, Adrian Stelmasiak, Andrzej Poltorak

**Affiliations:** Department of Technique and Food Development, Institute of Human Nutrition Sciences, Warsaw University of Life Sciences, Nowoursynowska 159c Street, 32, 02-776 Warsaw, Poland; kolodziejczakklaudia@gmail.com (K.K.); monika_marcinkowska_lesiak@sggw.edu.pl (M.M.-L.); iwona_wojtasik_kalinowska@sggw.edu.pl (I.W.-K.); arkadiusz_szpicer@sggw.edu.pl (A.S.); adrian_stelmasiak@sggw.edu.pl (A.S.); andrzej_poltorak@sggw.edu.pl (A.P.)

**Keywords:** grilled pork, marinades, antioxidant activity, polycyclic aromatic hydrocarbons

## Abstract

Marinating is one of the most common methods of pre-processing meat. Appropriate selection of marinade ingredients can influence the physicochemical properties of the meat and can reduce the level of polycyclic aromatic hydrocarbons (PAHs) in the final product. The effects of the inclusion of natural plant extracts such as bay leaf (BL), black pepper (BP), turmeric (TU), jalapeno pepper (JP) and tamarind paste (TA) in marinades on the physicochemical properties of grilled pork neck were studied. The addition of spice extracts to marinades increased the proportion of colour components L* and b*. The use of TU, TA, JP, MX and C marinades lowered the hardness and pH of the meat. The highest phenolic compound levels were observed in the case of the mixture of all extracts (MX) and JP marinades, and the highest total antioxidant capacity was exhibited by the BL and MX marinades. The highest PAH content was recorded in the CON marinade (Σ12PAH 98.48 ± 0.81 µg/kg) and the lowest in the JP marinade (4.76 ± 0.08 µg/kg), which had the strongest, statistically significant reducing effect (95% reduction) on PAH levels. Analysis of correlation coefficients showed a relationship between the total antioxidant capacity of the marinades and the PAH content in grilled pork.

## 1. Introduction

The grilling of meats is a popular method of heat treatment among consumers [1], where the raw or semi-processed product is placed over a heat source and subjected to high temperatures until the desired quality is achieved [2]. However, grilling is associated with the formation of by-products such as polycyclic aromatic hydrocarbons (PAHs), which are formed via pyrolysis and pyrosynthesis processes [3]. PAHs are thought to be formed by three mechanisms that take place during the thermal processing of food: pyrolysis of organic matter (protein, fat), leakage of cellular juice onto the heat source and incomplete combustion of the fuel [4]. During these processes, carbon and hydrogen atoms combine, generating single or double bonds and, in turn, cyclic compounds. However, the exact course of these reactions is unknown; PAHs are presumed to be formed by complex processes such as hydrogen abstraction and acetylene addition (HACA) mechanisms, or from radicals or the Diels–Alder mechanism [5,6]. The formed PAH molecules are transported upwards with the smoke, where they are deposited on the surface of the heat-treated product [4,7,8].

PAHs constitute a group of organic environmental and food pollutants. PAHs contaminate both raw materials and processed products. In raw materials, PAHs mainly originate from the environment, where they are deposited with water or air, e.g., on the surfaces of vegetables or fruit. PAHs can also be ingested by livestock through water or contaminated feed. However, greater exposure to PAHs is associated with the consumption of foods in which these compounds were formed as a by-product of heat treatment [9]. Examples of such foods include sausages, fish, dairy products, vegetable oils, coffee and tea [10]. The size and shape of PAHs determine their physicochemical properties and biological activity. Based on the number of rings in the molecule, PAHs are divided into two groups: light PAHs, whose molecule consists of between two and four rings, and heavy PAHs, whose molecule is made up of five or more rings [9,11,12]. Compounds in the light category have relatively low toxicity and high volatility. However, heavy PAHs are more stable and pose a greater risk to human health [13,14]. Examples of compounds in the heavy PAH group include fluorene (F), anthracene (ANT), fluoranthene (FL), benz[a]anthracene (BaA), chrysene (CHR). In contrast, the group of heavy PAHs includes, among others, benzo[a]pyrene (BaP), benzo[b]fluoranthene (BbFL), benzo[g,h,i]perylene (BghiP), benzo[k]fluoranthene (BkF), dibenz[a,h]anthracene (DBahA) and indeno[1,2,3-cd]pyrene (IP) [10]. Additionally, PAHs dissolve well in organic solvents and are lipo- and lithophilic, which affects their distribution in the environment and bioaccumulation in living organisms [9].

PAHs ingested with contaminated food are metabolised in the body after absorption, which increases the polarity of PAH molecules, and consequently are removed from the body more rapidly [9]. However, such products display higher toxicity than the original molecule [9,14]. Numerous studies have confirmed the toxic, genotoxic, mutagenic and carcinogenic effects of PAHs on the human body [10,13,15]. Therefore, it is important to monitor PAH levels in food and discover solutions to reduce their concentrations in food products [16].

In general, grilling leads to PAH contamination of food. Factors that affect PAH levels in grilled meat include the grilling method, duration and temperature of the process, distance from the heat source and type of fuel used [17]. Pre-treatment of the raw material, fat content and post-process handling are also important [1,18]. One of the most popular methods of pre-treating grilled meat is marinating, which improves its sensory characteristics. Additionally, the appropriate choice of marinade ingredients can further influence PAH reduction in the product [19]. Wang et al. [19] investigated the possibility of decreasing PAH levels in grilled meat by using different types of tea to marinate chicken wings. Among six teas tested, three marinades, from green (81.99 µg/kg), white (132.08 µg/kg) and yellow (146.33 µg/kg) tea, significantly reduced the PAH concentration compared to the control sample (190.69 µg/kg). Green tea showed the greatest PAH content reduction among the samples tested. Furthermore, dark (293.79 µg/kg) and black (430.03 µg/kg) teas exhibited a PAH content increase in grilled wings. According to Wang et al. [19], the PAH content reduction stemmed from the phenolic compounds present in teas, which can scavenge free radicals and inhibit PAH-forming reactions. Wang et al. [1] showed the individual effects of eight phenolic compounds on the amount of PAHs formed in grilled meat. Quinic acid (6.45 µg/kg) reduced the PAH levels in the samples by more than half compared to the control (14.18 µg/kg). Additionally, a further increase in PAH content decline occurs with increasing phenolic compound concentration in the marinade. PAHs can be also reduced by marinating meat in vinegar, where white wine, red wine, apple cider, elderflower and apple cider with raspberry juice displayed significant PAH content reductions compared to the control sample (31.47 µg/kg). An 82% PAH content reduction occurs in elderberry vinegar (5.60 µg/kg) and 79% in white wine vinegar (6.67 µg/kg) [20]. Interestingly, Viegas et al. [21] investigated the effect of marinating pork in different types of beer, where a reduction in PAH formation in beer-marinated samples was observed compared to the control sample. Black beer showed the greatest effect, reducing the PAH content from 20.57 to 9.74 µg/kg. Non-alcoholic pilsner (15.50 µg/kg) was more effective than that containing alcohol (17.82 µg/kg). Important components of marinades are additives, which influence and enhance the taste of meat, especially spices. Eldaly et al. [22] investigated the effect of a yoghurt-based marinade on levels of five PAHs in grilled beef (kebab and kofta). In addition to yoghurt, individual ingredients such as salt, turmeric, curry, cardamom, vinegar, mustard and onion were examined. Unmarinated kebab contained 119.8 µg/kg of PAHs and kofta contained 59.2 µg/kg of PAHs. Marinating of meat before grilling reduced PAH levels to 57.93 µg/kg in grilled kebabs and 30.2 µg/kg in grilled kofta. Benzo[a]pyrene levels were reduced from 9.2 (unmarinated kebab) and 26.16 µg/kg (unmarinated kofta) to below the detection limit. The pH of the marinade can also significantly affect PAH formation in grilled products. Studies have shown that an alkaline pH increases the PAH content, particularly heavy PAHs, in grilled chicken meat [23]. Wongmaneepratip and Vangnai [23] showed that the addition of 15 (B—base marinade) or 30 g (DB—double-base marinade) of sodium bicarbonate to the marinade significantly increased the PAH content in grilled chicken (B—683.2 µg/kg; DB—1781.4 µg/kg) compared to the control sample marinated in palm oil (484.9 µg/kg). Farhadian et al. [24] studied PAH content in beef satay marinated in a basic marinade (B) and a basic marinade with oil (B-O), as well as modified marinades: basic with lemon juice (B-L), basic with oil and lemon juice (B-O-L) and basic with oil and tamarind juice (B-O-T). Raw and unmarinated grilled beef satay were also analysed, but did not show any evidence of PAHs. The lowest content of the three tested PAHs was recorded for satay marinated in the basic marinade (47.6 µg/kg) and the highest for satay in the basic marinade with added oil (109 µg/kg). Unmarinated satay contained significantly higher PAH content (76.0 µg/kg) than satay in marinade B, but lower than marinade B-O. In this case, the PAH reduction may be related to the presence of spices such as onions, turmeric, lemongrass, garlic, coriander and cinnamon, which have strong antioxidant effects and inhibit PAH formation reactions. The increase in PAH content in the B-O marinade may be due to the higher fat content, which may be converted to PAHs upon exposure to high temperatures. Researchers also analysed the effect of lemon juice and tamarind juice on the PAH content of beef satay, where the B-L marinade had lower PAH content (52.1 76.0 µg/kg) than unmarinated samples, but higher in B marinade. The addition of either juice resulted in significant PAH content compared to the B marinade with oil added. Beef satay in B-O-L marinade had a PAH content of 59.6 µg/kg, while the B-O-T marinade had a PAH content of 86.9 µg/kg. The addition of juices lowered the pH and, thus, inhibited PAH formation reactions [24]. 

The currently available literature data indicate that an appropriate marinade composition can reduce the risk of PAH formation in heat-treated meat products. At the same time, the use of marinade additives can affect the quality of meat in different ways. The aim of this study was to analyse the effect of the addition of four different plant extracts (bay leaf, black pepper, turmeric, jalapeno pepper) and tamarind paste to marinades on PAH content and physicochemical parameters, including colour, texture, basic composition, antioxidant activity and phenolic compound content, of grilled pork neck. The research conducted makes a great contribution to the field of agricultural science and the discipline of food and nutrition technology by contributing to the reduction of PAHs in grilled meat products. 

## 2. Materials and Methods

### 2.1. Sample Preparation and Grilling Procedure

Four types of natural plant extracts from the company Result (Poland) were used in the study: bay leaf (BL) extract, black pepper (BP) extract, turmeric (TU) extract, jalapeno pepper (JP) extract and tamarind (TA) paste (Surre, Thailand). A commercial (C) pork marinade from Knorr (Poland) and a mixture of all tested extracts (synergy effect) were also used to compare the effects of the individual marinades. The content of the different extracts added to the marinades and their sources are shown in Table 1. To each of the marinades, 5% of NaCl was added. Information on the extracts was presented in Table 2.

The pork neck was purchased from the local Wierzejki Meat Plant in Poland and cut into steaks 2.0 cm thick and weighing approx. 150 g. The meat was marinated for 24 h at 4 °C. The meat to marinade ratio was 1:1 (g/mL) according to the methodology described by Wang et al. [19]. After 24 h, the samples were removed from the marinades and grilled using a Weber Master-Touch Premium GBS E-577 grill. Temperature monitoring was carried out during the heat treatment using a digital meter (Testo 926, Lenzkirch, Germany). The charcoal temperature was between 280 and 300 °C. During grilling (12 min), all samples were turned over once every 2 min. The charcoal was replaced with new charcoal between the grilling of the different study groups. In accordance with the methodology described by Cordeiro et al. [20], once the geometric centre reached a minimum temperature of 75 °C, the samples were removed from the grill, cooled to room temperature and then vacuum-packed in polyethylene bags (Cryovac ^®^ VS26, Sealed Air Corporation, Elmwood Park, NJ, USA) and stored at 4 °C. pH, basic composition, textural properties, colour according to L*a*b* system, 12 PAHs and volatile compound profile were determined for all samples. The grilling process was carried out on a total of 64 pork neck steaks: 8 independent samples for each of the 8 test groups (control, samples marinated in aqueous solutions containing different plant extracts, samples marinated with a mixture of all extracts, samples marinated with a commercial marinade). 

### 2.2. Chemical Composition Analysis

Analysis of the basic composition was carried out using NIRFlex Solids N-500 in the spectral range of 12500–4000 cm^−1^ (BÜCHI Labortechnik GmbH, Essen, Germany). The measurement method consisted of scanning the meat sample three times in the measuring module of the spectrometer and averaging the obtained results for the percentages of water, protein, fat, ash and connective tissue [25].

### 2.3. pH Measurement

The pH value of the meat was measured using a Testo 205 pH meter with a penetration probe and automatic temperature compensation (Testo, Inc., Sparta, NJ, USA). The result was calculated as the arithmetic mean of three measurements. Calibration of the pH electrode was carried out with standardised buffers of pH 4.00 and 7.00 [26].

### 2.4. Colour Measurement

The colour of the grilled pork neck was measured using a CR-400 chroma meter (Konica Minolta, Inc., Tokyo, Japan) and standardised with a white plate (L* = 98.45, a* = −0.10, b* = −0.13). Ten measurements each were taken on the surface and cross-section of the grilled pork neck of the 8 study groups. The colour values were expressed according to the Commission International de l’Eclairage system (CIE, 1976) and presented as CIE L* (lightness), CIE a* (redness) and CIE b* (yellowness).

### 2.5. Texture Measurement

Texture profile analysis (TPA) was carried out using the Instron 5965 Universal Testing Machine (Instron, Norwood, MA, USA). Compression of the samples was achieved using a flat, 4 cm diameter cylinder probe. Samples measuring 18 mm in height and 18 mm in diameter were double-compressed to the point of 50% reduction in their initial height with a relaxation time of 3 s (cell capacity—500 N; head speed—2 mm/min). Based on the force versus time curve, hardness (N/cm^2^), springiness (-), cohesiveness (J/cm^2^), gumminess (N) and chewiness (N) were determined. The measurement was conducted in six repetitions for each sample [27].

### 2.6. Determination of the Radical Scavenging of Meat Samples by DPPH Assay

Preparation of ethanolic extracts: 2.5 g pork neck was mixed with 7.5 mL of ethanol and homogenised for 2 min at 14 rpm × 1000 (Ultra Turrax homogeniser, IKA T18 basic, Germany). Samples were extracted at room temperature for 15 min using a rotary shaker (MyLab SLRM-3, NanoEnTek Inc., Seoul, Korea) and then centrifuged for 15 min at 18,000 rpm (MPW-251, MPW Med. Instruments, Warszawa, Poland). The supernatant was decanted from the precipitate and the antioxidant activity and total phenolic content were tested.

The antiradical activity of the analysed pork necks was measured as the reducing capacity with respect to 1,1-diphenyl-2-picrylhydrazyl (DPPH) radical [28]. For this purpose, 0.5 mL ethanolic extract was added to 3.5 mL ethanolic DPPH solution (0.1 mM). The mixture was stirred for 30 s and allowed to stand for 30 min in the dark. Absorbance was measured at 517 nm (Tecan Spark^TM^ 10M, Männedorf, Switzerland). Ethanol of 80 g/L was used as reference solution. A control sample was prepared without the addition of extract (the extract was replaced with 80 g/L ethanol). Measurements were carried out in triplicate. The total antioxidant activity (TAA) was expressed as % reduction in DPPH radical and calculated using the following formula:

TAA(%) = ((Abs_sample_ − Abs_control_)/Abs_sample_) × 100%

where TAA is the total antioxidant activity (%) and Abs is the absorbance at 517 nm wavelength.

### 2.7. Determination of the Total Phenolic Content (TPC) in Meat Samples

The sum of phenolic compounds in pork neck was determined using the Folin–Ciocalteu method described by Singleton and Rossi [29], with modifications. First, 6.0 mL distilled water and 0.5 mL Folin and Ciocalteu reagent (Sigma Aldrich Inc., St. Louis, MA, USA) were added to 0.1 mL ethanolic extract. After 3 min, 1.5 mL sodium carbonate solution (7.5% *w*/*v*) (Sigma Aldrich Inc., St. Louis, MA, USA) was added, and water was added up to 10 mL. The reaction mixture was maintained for 30 min in a water bath (WNB 7 Memmert, Germany) at 40 °C. Absorbance was measured spectrophotometrically at 765 nm (Tecan Spark^TM^ 10M, Männedorf, Switzerland). The sum of the phenolic compounds was expressed as gallic acid equivalents (GAE) based on a pre-prepared standard curve. The results were presented as the mean of three replicates in mg of gallic acid per 100 g sample (mg of GAE/100g).

### 2.8. Standards and Calibration Solution

**Figure 1:**
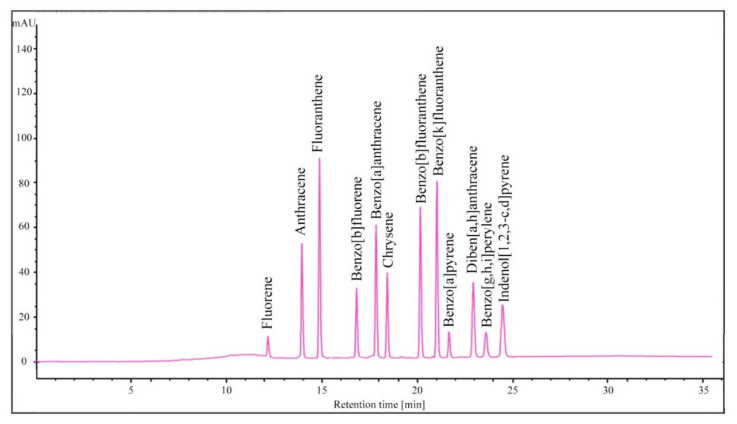
Chromatogram of the corresponding 12 PAH standards: fluorene, anthracene, fluoranthene, benzo[b]fluorene, benz[a]anthracene, chrysene, benzo[b]fluoranthene, benzo[k]fluoranthene, benza[a]pyrene, diben[a,h]anthracene, benzo[g,h,i]perylene and indeno[1,2,3-cd]pyrene.

The standards of fluorene (F), anthracene (ANT), fluoranthene (FL), benzo[b]fluorene (BbF), benz[a]anthracene (BaA), chrysene (CHR), benzo[b]fluoranthene (BbFL), benzo[k]fluoranthene (BkF), benza[a]pyrene (BaP), diben[a,h]anthracene (DBahA), benzo[g,h,i]perylene (BghiP) and indeno[1,2,3-cd]pyrene (IP) were obtained from Perlan Agilent Technologies, USA. Stock solutions of each PAH were prepared by dissolving 0.01 g of each standard in high-purity acetonitrile (Sigma-Aldrich, Darmstadt, Germany). Calibration solutions were then prepared via a series of dilutions of the stock solution into a five-point concentration range of 0.05–20.0 µg/kg. Calibration curves were made using the external standard method and plotted as a linear dependence of the measured signal as a function of the peak area of the standard solution. A chromatogram showing the peaks of the 12 PAHs is shown in Figure 1.

### 2.9. PAH Extraction and Quantification

PAH extraction was performed according to the method described by Bogdanović et al. [30]. The first step involved the lipid saponification reaction via incubation of 10 g homogenised samples in 1 M ethanolic potassium hydroxide solution (25 mL) in a water bath (WNB 7 Memmert, Germany) at 80 °C for 2 h. Samples were then transferred to a separating funnel and extracted three times with 15 mL cyclohexane, which was then evaporated. The next step consisted of two-step sample purification using the SPE method described in detail Kafouris et al. [31]. Purification was conducted using a C-18 column, which was activated with methanol (24 mL) and acetonitrile (24 mL). A flask with the oily residue from the evaporation of cyclohexane was washed 3 times with a mixture of acetone/acetonitrile, 40/60, (1 mL), which was transferred to a centrifuge tube. After centrifugation at 5000 rpm for 5 min, the supernatant liquid was passed through an activated C-18 column and the eluant collected. The eluant was washed with cyclohexane (2 mL) followed by acetone/acetonitrile mixture (40/60, 3 × 1 mL). The resulting solution was centrifuged and the studied fluid was passed through an activated C-18 column and collected. The column was then washed with acetone/acetonitrile mixture (40/60, 5 mL) and the eluant collected, which was evaporated to dryness using a rotary evaporator (ROTAVAPOR R-100 Büchi, Switzerland). The residue was dissolved in hexane (1 mL) and purified using a Florisil SPE column, which was first activated with dichloromethane (15 mL) and hexane (12 mL). The flask containing the sample was washed with dichloromethane/hexane, 25/75 (3 × 1 mL), and the obtained solution was passed through a column and collected. The column was then washed with dichloromethane/hexane (5 mL), the eluant collected and the solvent evaporated using a rotary evaporator. The residue was dissolved in acetonitrile (1.2 mL) and filtered through a 0.45 µm membrane syringe filter.

Determination of 12 PAHs was performed via high-performance liquid chromatography with a fluorescence detector (Analytical HPLC, 1260 Infinity II LC System, Agilent). Separation was performed on an appropriately sized Agilent ZORBAX Eclipse PAH column 4.6 × 150 mm, 3.5 µm, flow rate 1.3 mL/min. The mobile phase consisted of water (A) and acetonitrile (B), column temperature of 25 °C, injection volume of 5 µL. The separation parameters are listed in Table 3.

### 2.10. Recovery Studies

The measurement procedure consisted of the preparation and analysis of blank samples, samples with unknown PAH concentrations and two samples containing calibration solutions (2.00 and 20.00 µg/kg). The design of the experiment was based on previous studies described in detail by Onopiuk et al. [32]. To prevent matrix effects on peak positions in the chromatogram, and to assess the percentage recovery of PAHs, unenriched and enriched samples were analysed under the same conditions.

For each of the calibration curves of the 12 PAHs, coefficients of variation for the concentration limits were calculated and then the correlation coefficient (r) was calculated. Limit of detection (LOD) and limit of quantification (LOQ) were calculated using the following formulas, respectively: Cm + 3SD and Cm + 6SD, where Cm was the mean value of PAH concentration in the blank and SD was the standard deviation [16]. Recovery values were determined by performing the entire analytical procedure for the determination of PAHs in meat samples enriched with a mixture of standards (mix F, ANT, FL, BbF, BaA, CHR, BbFL, BkF, BaP, DBahA, BghiP, IP) at concentrations of 2.00 and 20.00 µg/kg in triplicate. In addition, in order to determine the PAH content in the sample matrix, the procedure for PAH determination in unenriched and blank samples was performed. Recovery values were calculated from the obtained results (Table 4).

### 2.11. Analysis of Volatile Compound Profile

Volatile compounds in meat samples were determined using an electronic nose—Heracles II (Alpha M.O.S., Toulouse, France). This method was previously described by Wojtasik-Kalinowska et al. [28]. The Kovats indexes were determined based on alkane standards (*n*-butane to *n*-hexadecane) (Restek) measured under the same conditions as the sample. Identification of the volatile compounds was conducted using AroChemBase (Alpha MOS Co., Toulouse, France). A 3 g sample was placed in a 20 mL headspace vial and capped with a Teflon-faced silicon rubber cap. Then, the vials were incubated at 55 °C for 900 s under agitation speed (8.33 Hz). Carrier gas (hydrogen) was circulated at a constant flow rate (1 mL min^−1^). The injector temperature was 200 °C, injected volume was 2500 µL and injection speed 125 mL s^−1^. The analytes were collected in the trap at 15 °C and then divided and simultaneously transferred to the two columns. A carrier gas was applied at a constant pressure of 80 kPa. The split flow rate was 10 mL min^−1^ at the column heads. Samples were repeatedly analysed five times.

### 2.12. Statistical Analysis

The data were statistically analysed using the Statistica 13.1 program (StatSoft Inc., Tulsa, OK, USA). One-way analysis of variance was performed using Tukey’s test at a level of significance of *p* < 0.05. The results were presented as mean values with their standard deviation (SD). The flavour profile was carried out using principal component analysis (PCA), AlphaSoft Version 8.0.

## 3. Results and Discussion

### 3.1. Chemical Composition of Grilled Meat

Meat samples from the CON group, marinated using natural extracts of herbs and spices, and the C marinade were subjected to basic composition analysis (Table 5). The BP (49.15 ± 0.17%) and TA (49.55 ± 0.36%) marinades (*p* < 0.05) showed the statistically significantly lowest water content. In contrast, the TU (55.16 ± 0.32%) and JP (55.01 ± 0.13%) marinades had the highest moisture content. The moisture measurement results of the samples were in agreement with literature data. García-Lomillo et al. [33] reported 57.3% moisture content for meat marinated in red wine pomace seasoning. Slightly higher results were reported by O’Neil et al. [34] for pork chops marinated in piri marinade. Depending on the type of treatment used (high pressure and griddle or steam cooking), the moisture content of the samples ranged from 61.87 to 65.23%.

The highest statistically significant (*p* < 0.05) fat content was found for samples marinated with BP (21.78 ± 0.08%) and MX (21.59 ± 0.34%), whereas the lowest was found for the JP (16.43 ± 0.05%) and TU (16.47 ± 0.41%) marinades. The TA marinade displayed the statistically highest protein content (27.62 ± 0.11%) among the samples. The lowest protein content was found for the C (22.52 ± 0.15%) and MX marinades (22.58 ± 0.15%). These results were slightly lower than the values reported by O’Neill et al. [34], which ranged between 29.08 and 30.31% and could be due to the meat type used (pork loin) and the treatment method. The MX marinade showed lowest salt content compared to the other samples (1.34 ± 0.18%), whereas the C marinade had the highest salt content (2.77 ± 0.03%). This result was statistically significant (*p* > 0.05) in relation to the other study groups. The connective tissue content ranged from 2.84% for BL to 3.78% for MX.

### 3.2. pH of Marinated Meat

The pH of the marinated meat was measured (Table 5), where the majority of samples showed a neutral or acidic pH. The lowest pH value was recorded for the TA marinade (2.31 ± 0.06). The statistically significantly highest (*p* < 0.05), and near neutral, pH values were recorded for the BL (7.02 ± 0.01) and BP (7.00 ± 0.01) marinades. Similar results were reported by Mozuriene et al. [35], where the pH value of meat ranged from 5.06 to 6.70. Park et al. [2] also found a slightly acidic pH in marinated pork. In their study, pork belly was marinated in green tea and yerba mate at different concentrations. The samples tested had pH values in the range of 5.75–6.04, which were similar to those obtained in our study.

### 3.3. Colour Measurement

Grilled pork samples were subjected to instrumental colour analysis using the L*a*b* system. Measurements were carried out on the surfaces and cross-sections of the meat samples (Table 6). The BL marinade (L* 46.94 ± 2.71) had the highest value of the colour component L*, whereas the C marinade had the lowest lightness value (L* 40.14 ± 2.11). Similar values of the L* parameter were reported by Mozuriene et al. [35], in which the lightness of pork samples was shown to be in the range of 45.63–65.99 depending on the meat type (pork neck, shoulder, ham muscle, M. *longissimus dorsi*, loin) and marinade type (*P. acidilactici*, *P. pentosaceus*, *L. sakei*). O’Neill et al. [34] also reported similar values for the L* colour component, ranging between 45.58 and 59.34.

The highest level of the a* component among all the samples was recorded for the BP marinade (a* 11.60 ± 0.93). This value was not statistically significantly different (*p* > 0.05) compared to the TA (a* 11.14 ± 0.96), JP (a* 11.02 ± 1.21) or TU (a* 10.25 ± 0.86) marinades. The BL marinade had the lowest red colour intensity (a* 8.79 ± 0.59). Studies by Mozuriene et al. [35] showed similar levels of the a* component for pork, with values ranging from 3.54 to 14.01. Moreover, O’Neill et al. [34] showed similar a* component values ranging from 7.45 to 9.33. The lowest intensity of yellow colour was recorded for the MX marinade (b* = 14.02). All other samples did not show a statistically significant difference (*p* > 0.05), except for the TU marinade, which had the statistically significantly (*p* < 0.05) highest proportion of yellow colour (b* = 20.60), which resulted directly from its characteristic yellow colour.

We observed that the BL (68.26 ± 1.55) and BP (67.62 ± 2.21) marinades had the highest, statistically significant (*p* < 0.05) level of the L* colour component. The CON marinade (L* 61.90 ± 2.48) had the lowest L* component, in contrast to the surface colour test. The highest intensity of red colour was recorded for the TU (a* 10.48 ± 0.76) and JP (a* 10.31 ± 0.91) marinades. The C group (a* 10.03 ± 1.28) and JP marinade (a* 9.35 ± 0.80) were not statistically significantly different (*p* > 0.05). The JP marinade displayed the least red (a* 8.22 ± 0.83), but belonged to the same statistical group as BL, TA, MX and C. The MX marinade had the highest b* component (b* 10.32 ± 0.50). The C marinade had the lowest intensity of yellow colour (b* 8.21 ± 0.60). The addition of spice extracts to the marinade increased the proportion of the L* and b* components in each case. For the a* component, samples marinated with extracts had a lower proportion of this component compared to C samples, except for the TU and JP marinades.

### 3.4. Texture Measurement

Grilled meat samples were subjected to instrumental texture analysis (Table 6), where the CON group had the highest statistically significant (*p* < 0.05) hardness (79.84 ± 9.84), and the BL, TU, and MX marinades were in the same statistical group. The BP marinade had the lowest hardness (60.22 ± 6.29). The C marinade displayed the lowest springiness value (0.24 ± 0.03), whereas the BL marinade had a slightly higher value (0.30 ± 0.06). Additionally, the BP marinade had the most springiness (0.48 ± 0.02), but this was not statistically significantly different compared to the JP (0.45 ± 0.02) and MX (0.44 ± 0.01) marinades. The cohesiveness value of the samples ranged from 0.29 (BL) to 0.44 (BP). In the case of gumminess, the BL marinade had the lowest values (20.14 ± 1.86), whereas the JP marinade had the highest gumminess and chewiness (gumminess 31.60 ± 2.29, chewiness 14.29 ± 0.48). Among all samples, the C marinade possessed the lowest chewiness (5.32 ± 1.60), which was statistically significantly different compared to the other samples (*p* < 0.05). Similar texture measurement results were reported by Żochowska-Kujawska et al. [36], where the hardness and cohesiveness values of marinated meat were in the ranges of 41.77–74.32 and 0.37–0.46, respectively. Furthermore, Żochowska-Kujawska et al. [36] showed that the springiness of meat samples was between 0.87 and 1.13, compared to our results; differences in this parameter may stem from the type of marinade ingredients (red dry wine, kefir, lemon juice, raw pineapple juice).

### 3.5. DPPH Scavenging Activities and TPC of Meat Samples

**Figure 2:**
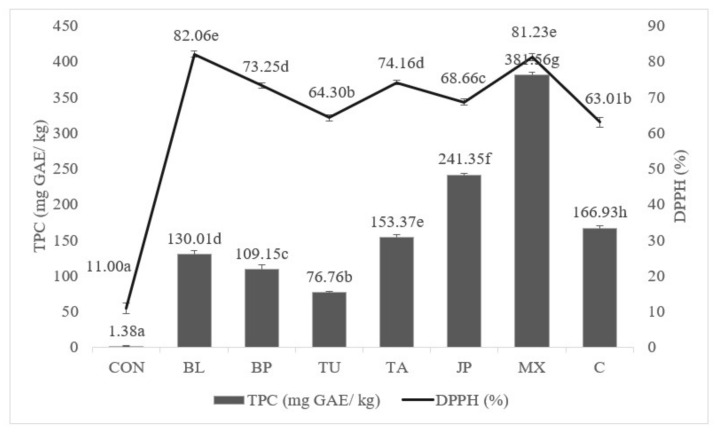
The effect of marinade on TPC and DPPH (mean ± standard deviation) of grilled pork. a–h—the mean values marked with various lowercase letters correspond to statistically significant differences (*p* < 0.05).

Analysis of the TPC of the grilled pork meat (Figure 2) revealed that the addition of vegetable extracts and TA to the marinades had a statistically significant (*p* < 0.05) effect, resulting in increased phenolic content. The lowest TPC was recorded for the CON group (1.38 ± 0.47 mg GAE/kg). Among the tested marinades, the TA marinade displayed the lowest TPC (76.76 ± 0.98 mg GAE/kg), while relatively high TPC was found in the C marinade (166.93 ± 3.62 mg GAE/kg). The MX marinade possessed the highest TPC (381.56 ± 4.08 mg GAE/kg). Among the individually added extracts, the highest TPC level of 241.35 ± 2.34 mg GAE/kg meat was observed for the JP marinade.

Marinated pork samples were tested for total antioxidant capacity using synthetic radical DPPH (Figure 2). The lowest antioxidant capacity was observed for C samples (11 ± 1.46%), whose values were statistically significantly different from those of the other samples (*p* < 0.05). The BP and TA marinades had similar antioxidant capacity (BP 73.25 ± 0.81%, TA 74.16 ± 0.69%). The BL (82.06 ± 0.90%) and MX (81.23 ± 0.94%) marinades showed the highest total antioxidant capacity.

### 3.6. Effect of Marinades on PAH Formation

Quantitative chromatographic analysis of the PAH content in the marinated and grilled meat samples was performed (Table 7). The highest PAH content was recorded for the CON marinade (Σ12PAH 98.48 ± 0.81 µg/kg). Lower values of total PAHs by 37% were found for samples in the commercial marinade (61.56 ± 0.66 µg/kg), and, of the samples marinated with extracts, the BP samples had the highest levels of PAHs (45.95 ± 1.02 µg/kg). The lowest PAH content was found in the JP marinade, where the total PAH content of all tested compounds was 4.76 ± 0.08 µg/kg, and this value was 95% lower than in the control marinade. In the remaining samples, PAH levels had a range of 21.43–41.66 µg/kg (58–78% reduction). The highest levels of light PAHs were found in the CON marinade (54.58 ± 1.11 µg/kg); a 7% lower value was observed for the C marinade (50.98 ± 0.66 µg/kg), whereas the BP marinade had the highest value (43.16 ± 0.99 µg/kg). In JP marinade samples, both light (3.92 ± 0.11 µg/kg) and heavy (0.84 ± 0.06 µg/kg) PAH content was the lowest among all tested samples. It also differed from the control group by 93% for light PAHs and 98% for heavy PAHs. The highest PAH level from the heavy group was observed in the CON group (43.90 ± 0.95 µg/kg).

Analysis of individual light PAH content showed that anthracene was detected in all samples. The BP and C marinades possessed the highest amount of anthracene (5.14 ± 0.17 and 3.12 ± 0.10 µg/kg, respectively), whereas the BL and MX marinades had the lowest (0.35 ± 0.04 µg/kg). BaA levels were highest in the CON group (3.18 ± 0.17 µg/kg) and BP marinade (2.22 ± 0.08 µg/kg). The lowest BaA and BbF content were recorded in the JP marinade, where BaA was 0.20 ± 0.01 µg/kg (94% reduction) and BbF was 2.90 ± 0.11 µg/kg (90% reduction). BbA was not detected in the TA marinade. The highest BbF content was found in the CON group (29.57 ± 0.45 µg/kg) and C marinade (22.39 ± 0.81 µg/kg). High amounts of CHR were present in the CON group (18.79 ± 1.05 µg/kg) and C marinade (18.97 ± 0.29 µg/kg). However, CHR was not detected in the JP marinade. The MX marinade (0.70 ± 0.02 µg/kg) had the lowest CHR content, which was 96% lower than in the control group. The least fluoranthene was observed for the JP marinade (0.28 ± 0.02 µg/kg) and the highest was observed in BL (5.93 ± 0.37 µg/kg) and C (3.18 ± 0.05 µg/kg) marinades. Samples in the BL marinade had the lowest level of fluorene (0.33 ± 0.01 µg/kg); in contrast, none was detected in the TU, TA, JP and MX marinades. The highest level of fluorene was observed in the C marinade (1.88 ± 0.04 µg/kg).

BaP, which belongs to the group of heavy PAHs, was not detected only in samples with the JP marinade. The highest levels of this compound were detected in the CON group (1.70 ± 0.08 µg/kg) and C (1.66 ± 0.06 µg/kg) marinade, which was the case for most other heavy PAHs. BbFL was absent in the BL marinade, but, in the C marinade it was found in the highest amount (1.47 ± 0.10 µg/kg), and in the TA marinade, it was observed in the lowest amount, reduced by 89% (0.13 ± 0.01 µg/kg). BghiP, which was absent in TU, was present in high amounts in the C marinade (1.70 ± 0.03 µg/kg) and CON (0.79 ± 0.03 µg/kg) marinades. In the other samples, its content ranged from 0.43 to 0.45 µg/kg (43–46% reduction). BkF was only detected in marinades CON (1.47 ± 0.15 µg/kg), C (3.04 ± 0.02 µg/kg) and BP (0.14 ± 0.03 µg/kg), where the use of the marinade allowed a 90% reduction in the content of this compound. The highest level of DBahA was found in the CON group (38.14 ± 0.75 µg/kg) and the lowest in the BP marinade (0.51 ± 0.03 µg/kg). IP was not detected in any of the marinated meat samples tested.

The results obtained were compared with the limits defined in Regulation 1881/2006. In all samples, the level of BaP was below 2 µg, the highest admissible by Regulation 1881/2006 for this compound in food. The highest admissible level of the content of 4PAH (bezno[a]pyrene, chrysene, benzo[a]anthracene, bezno[b]fluoranthene), which is a relevant indicator of PAH content in food, is 12 µg. Only three samples exceeded this limit; these were samples in the control marinade, with black pepper extract, and in the market marinade.

Numerous studies have reported the effects of marinades on PAH levels in grilled meat. Farhadian et al. [24] investigated the effect of adding oil, lemon juice or TA to a base marinade on PAH levels in grilled beef. The base marinade included sugar, water, onion, TU, lemongrass, salt, garlic, coriander and cinnamon. BaP, BbFL and fluoranthene levels were determined, where BaP levels in meat samples grilled for 12 min ranged from 1.20 to 4.08 µg/kg. These results are comparable to those obtained our study. Farhadian et al. [24] calculated the sum of PAH content according to base marinade (B), base marinade with oil (B-O), lemon juice (B-L), oil and lemon juice (B-O-L) and TA (B-O-T), which were 47.6 µg/kg, 109 µg/kg, 52.1 µg/kg, 59.6 µg/kg and 86.9 µg/kg, respectively. Wongmaneepratip and Vangnai [23] showed that the PAH content of chicken meat marinated in a control marinade (water, sugar, oyster sauce, salt, garlic, pepper) was the sum of four PAHs, 26.3 µg/kg, and the sum of 16 PAHs, 190.1 µg/kg. In the present study, the sum of four PAHs was in the range of 0.61–24.82 µg/kg. These results are comparable to those reported by Wongmaneepratip and Vangnai [23] for CON and C marinades. Viegas et al. [21] examined PAH content in grilled pork unmarinated (C) or marinated in different types of beer (pilsner beer—PBp, non-alcoholic pilsner beer—P0Bp and black beer—BBp) and found slightly higher results for most PAHs, e.g., BaP content was between 1.07 and 2.71 µg/kg and BbFL 1.42–3.23 µg/kg. The sum value of the eight tested PAHs ranged from 9.74 (BBp) to 20.57 (C) µg/kg. Nevertheless, the PAH content of grilled meat depends on many factors. A study by Wongmaneepratip and Vangnai [23] analysed chicken breast, which has different characteristics from the pork used in this study. The marinade composition, grilling parameters and sample dimensions were also different. In the study by Farhadian et al. [24], beef was grilled, the process temperature was unknown, and the marinade composition was significantly different from the marinades used in the current study. Similar relationships between the use of an appropriate marinade and the reduction of PAH levels in the product have been observed, but an exact comparison of the results is not possible due to the high variability of the determinants of PAH content in grilled meat (meat type, marinade composition, heat treatment parameters).

The JP marinade’s lowest PAH content was due to JP being a valuable source of carotenoids and capsaicin, both of which exhibit high free radical quenching capacity, as indicated by Lu et al. [14]. Vitaglione and Fogliano [37] and Janoszka [38] confirmed that the addition of antioxidants to meat could trap free radicals that contribute to the formation of intermediate cyclic compounds, which contribute to the generation of PAHs. Indeed, the application of natural extracts of herbs and spices to heat-treated meat products is an effective means of reducing the levels of cyclic organic compounds belonging to benzene derivatives. Herb and spice extracts contained in marinades scavenge free radicals during both hydrocarbon fragmentation and aromatic compound cyclisation. Kafouris et al. [31] determined a strong positive relationship between fat content and PAH formation in meat products. Studies indicate that the phenolic compounds contained in spices have a selective effect on individual PAHs. This was also supported by Lu et al. [14], where an inhibitory effect of spices was observed on BaB content, while no effect on BaA formation was demonstrated.

Analysis of correlation coefficients (Table 8) showed that there was a significant negative correlation between total antioxidant capacity and the content of 12 PAHs in grilled meat (−0.779). This correlation indicated that the antioxidant compounds present in the plant extracts effectively reduced PAH formation. A particularly strong correlation between the value of total antioxidant capacity and PAH content was observed for DBahA (−0.879). A strong negative correlation was observed between total antioxidant capacity and the content of BaA (−0.660), BbFL (0.620), BbF (0.503) and CHR (0.643). However, no significant relationship was found between the DPPH value and the content of other PAHs. Total phenolic compounds affected the PAH content of the samples to a lesser extent than DPPH. The negative correlation coefficient between the TPC and the content of 12 PAHs was −0.576. The content of phenolic compounds was negatively correlated with PAH content such as BaA (−0.624), CHR (−0.639) and DBahA (0.574) in the samples.

The correlation coefficient between the total antioxidant value and total phenolic compound content, as well as the sum of PAH content related to individual heavy and light groups, was also examined. The antioxidant capacity was observed to inhibit the formation of PAHs from both groups to a noticeably higher extent than the total phenolic compound content. There was a significantly negative correlation between the content of heavy PAHs and total antioxidant capacity in the samples tested (−0.903).

The results showed that all the extracts were effective in inhibiting the formation of PAHs. The phenolic compounds present in marinades serve as inhibitors of PAH formation by acting as free radical quenchers or scavengers. Marinades can also act as barriers to block direct contact between meat and the heat source. The active structural domains characteristic of different types of compounds may exhibit different types of inhibitory activity. The structure of phenolic compounds is a key determinant of the free radical scavenging ability and affects the dynamics of the cyclisation reaction that produces PAHs. Inhibition by phenolic compounds occurs not only by the scavenging of free radicals but also by combining with PAH intermediates to inhibit the completion of the reaction.

### 3.7. Volatile Compound Profile of Grilled Meat

**Figure 3:**
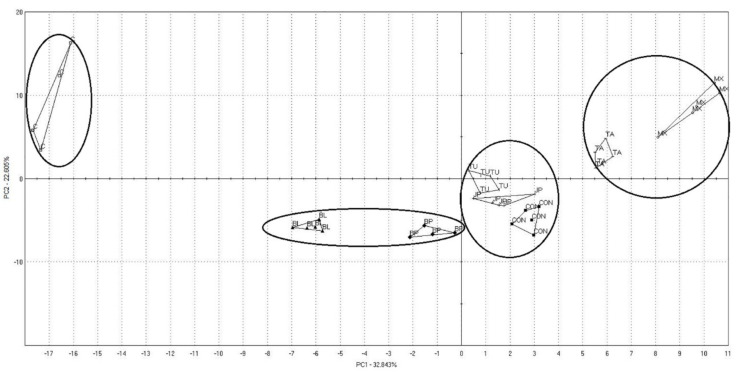
Volatile compounds identified in grilled pork meat samples.

Visualisation of the results of variance analysis is presented on a scores plot (Figure 3). PCA indicated the differences in the volatile composition of the eight analysed groups. The values of 32.8% data variance were explained by the vertical axis and 22.6% intercepted by the horizontal axis. The volatile compound profile of the CON group was in the same area as the TU and JP groups. The second set was the BL and BP groups and the third the TA and MX groups, which suggested similar aromas in these groups. However, the C group was in a separate area, which indicated that their composition was different from the other groups.

## 4. Conclusions

Grilling can lead to contamination of food with PAHs, which have toxic, mutagenic and carcinogenic effects. Therefore, it is important to explore solutions to reduce the PAH levels in food products. The formation of PAHs in grilled meat products is mainly attributed to the thermal degradation of organic matter, especially fats and amino acids. Thermal drip components, as a result of direct contact with heated fuel, undergo pyrolysis, which results in increased levels of PAHs in the resulting smoke. Our results showed that all the extracts were effective in inhibiting the formation of PAHs. The phenolic com-pounds in the marinades, especially the JP marinade, acted as inhibitors of PAH formation by quenching or scavenging free radicals. Analysis of correlation coefficients showed that there is a significant effect between total antioxidant capacity and the content of 12 PAHs in the grilled meat. The herb and spice extracts used in the marinades improved the meat quality by affecting the pH, textural properties, colour, PAH profile and volatile compounds in grilled pork neck. The natural antioxidants contained in the extracts can contribute to the inhibition of cyclisation and oxidation reactions, thus increasing the safety and shelf life of grilled meat products.

## Figures and Tables

**Table 1:** Type and amount of marinade extracts, NaCl content and names of study groups.

Type of Marinade	Latin Plant Name	Source of Extract/Paste	Sample Code	NaCl Content (%)	Extract/Paste Content (g/kg)
Pork neck, unmarinated	-	-	CON	5.0	0.0
Marinade with bay leaf extract	*Laurus nobilis*	Result, Poland	BL	5.0	6.0
Marinade with black pepper extract	*Piper nigrum*	Result, Poland	BP	5.0	6.0
Marinade with turmeric	*Curcuma longa*	Result, Poland	TU	5.0	6.0
Marinade with tamarind paste	*Tamarindus indica*	Suree, Thailand	TA	5.0	80.0
Marinade with jalapeno pepper extract	*Capsicum annuum*	Result, Poland	JP	5.0	6.0
Marinade with a mixture of all extracts	*-*	-	MX	5.0	a mixture of 100 mL of each marinade
Commercial marinade	*-*	Knorr, Poland	C	-	-

**Table 2:** Characteristics of plant extracts and tamarind paste.

Picture	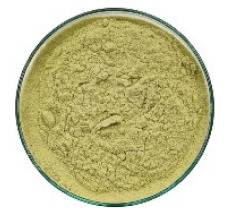	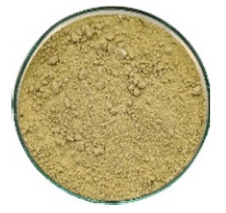	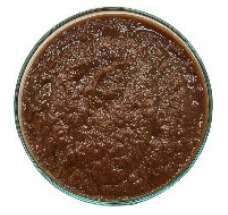	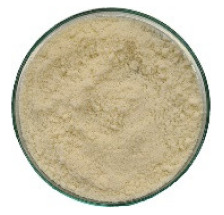	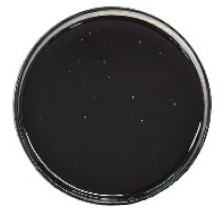	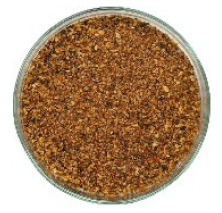
Extract	Bay leaf	Black pepper	Tamarind paste	Jalapeno peppers	Turmeric	Comercial seasoning for pork neck
Biologically active substances	Cyneol Geraniol	Piperine alkaloid	Tartaric acid	Capsaicin	Curcuminoids	No data
Concentration of biologically active substances ^a^	6 mg/100 g	42–43%	No data	6.6 ± 0.3%	7–10%	No data

^a^ information about the extracts is provided by the manufacturer.

**Table 3:** Separation parameters of HPLC/FLD method.

Chromatographic Conditions	
Parameter	Value
Analytical column	Agilent ZORBAX Eclipse PAH 4.6 × 150 mm, 3.5 µm
Column temperature	25 °C
Gradient	(A) Water, (B) Acetonitrile
	0 min, 40% B at 1.3 mL/min
	5 min, 40% B
	20 min, 100% B
	30 min, 100% B
	30.1 min, 40% B
	Post time 6 min
Injection volume	5.0 µL
Autosampler temperature	18 °C
Diode array detector	230 nm, band width 4 nm, reference 400 nm, reference band width 100 nm, 10 Hz
Fluorescence detector	Multisignal acquisition, set at λ_ex_ = 260 nm and λ_em_ = 350 nm (FLD A), 330 nm (FLD B), 440 nm (FLD C), 500 nm (FLD D), 18.51 Hz

**Table 4:** The method validation parameters.

PAH Compound	Linearity r^2^	Detection Limits LOD (µg/kg)	Detection Limits LOQ (µg/kg)	Recovery (%)	
				Level I (2.0 µg/kg)	Level II (20.00 µg/kg)
Fluorene F	0.9995	0.05	0.25	82.59	83.77
Anthracene ANT	0.9997	0.10	0.25	86.13	87.24
Fluoranthene FL	0.9998	0.10	0.25	90.39	92.38
Benzo[b]fluorene BbF	0.9995	0.05	0.25	85.73	86.26
Benz[a]anthracene BaA	0.9996	0.10	0.25	90.12	90.72
Chrysene CHR	0.9998	0.10	0.25	97.17	98.75
Benzo[a]pyrene BaP	0.9998	0.10	0.25	85.26	85.05
Benzo[b]fluoranthene BbFL	0.9998	0.10	0.25	91.69	93.12
Benzo[g,h,i]perylene BghiP	0.9995	0.05	0.25	83.15	86.51
Benzo[k]fluoranthene BkF	0.9996	0.10	0.25	92.44	94.76
Dibenz[a,h]anthracene DBahA	0.9995	0.05	0.25	95.27	96.18
Indeno[1,2,3-cd]pyrene IP	0.9994	0.10	0.25	81.13	81.29

**Table 5:** The effect of marinade on basic chemical composition and pH (mean ± standard deviation) of grilled pork.

Item	Group							
	CON	BL	BP	TU	TA	JP	MX	C
Moisture (%)	52.96 ^b^ ± 0.08	52.92 ^b^ ± 0.31	49.15 ^a^ ± 0.17	55.16 ^d^ ± 0.32	49.55 ^a^ ± 0.36	55.01 ^cd^ ± 0.13	52.54 ^b^ ± 0.72	54.09 ^c^ ± 0.16
Fat (%)	18.22 ^b^ ± 0.12	19.96 ^c^ ± 0.46	21.78 ^d^ ± 0.08	16.47 ^a^ ± 0.41	18.50 ^b^ ± 0.30	16.43 ^a^ ± 0.05	21.59 ^d^ ± 0.34	18.46 ^b^ ± 0.17
Protein (%)	24.48 ^c^ ± 0.24	23.63 ^b^ ± 0.05	25.23 ^d^ ± 0.06	24.64 ^c^ ± 0.20	27.62 ^e^ ± 0.11	23.94 ^b^ ± 0.14	22.58 ^a^ ± 0.15	22.52 ^a^ ± 0.15
Salt (%)	2.77 ^e^ ± 0.03	1.96 ^b^ ± 0.06	2.09 ^bc^ ± 0.04	2.23 ^cd^ ± 0.05	1.50 ^a^ ± 0.04	2.40 ^d^ ± 0.10	1.34 ^a^ ± 0.18	1.52 ^a^ ± 0.03
Connective Tissue (%)	3.31 ^bc^ ± 0.14	2.84 ^a^ ± 0.19	2.98 ^ab^ ± 0.07	3.53 ^cd^ ± 0.16	2.97 ^ab^ ± 0.08	3.32^bc^ ± 0.10	3.78 ^d^ ± 0.20	3.22 ^a^ ± 0.17
pH	6.96 ^f^ ± 0.03	7.02 ^f^ ± 0.01	7.00 ^f^ ± 0.01	6.83 ^e^ ± 0.01	2.31 ^a^ ± 0.06	4.55 ^b^ ± 0.03	5.58 ^c^ ± 0.06	6.02 ^d^ ± 0.03

CON—control (water + salt); BL—bay leaf; BP—black pepper; TU—turmeric; TA—tamarind paste; JP—jalapeno pepper; MX—all spice mix; C—commercial marinade.^a–f^—the mean values marked with various lowercase letters correspond to statistically significant differences within rows (*p* < 0.05).

**Table 6:** The effect of marinade on colour and texture parameters (mean ± standard deviation) of grilled pork.

Item	Group							
	CON	BL	BP	TU	TA	JP	MX	C
	Surface							
L*(%)	45.83 ^cd^ ± 1.74	46.94 ^d^ ± 2.71	41.91 ^ab^ ± 2.53	42.15 ^ab^ ± 1.50	41.91 ^ab^ ± 2.68	42.71 ^abc^ ± 2.69	43.88 ^bcd^ ± 1.86	40.14 ^a^ ± 2.11
a*(−)	8.86 ^ab^ ± 0.67	8.79 ^a^ ± 0.59	11.60 ^c^ ± 0.93	10.25 ^bc^ ± 0.86	11.14 ^c^ ± 0.96	11.02 ^c^ ± 1.21	9.00 ^ab^ ± 1.72	9.84 ^abc^ ± 0.98
b*(−)	15.31 ^a^ ± 1.32	14.77 ^a^ ± 1.08	15.72 ^a^ ± 1.54	20.60 ^b^ ± 1.40	15.12 ^a^ ± 1.38	15.41 ^a^ ± 1.42	14.02 ^a^ ± 2.33	15.86 ^a^ ± 1.72
	Cross-Section							
L*(%)	61.90 ^a^ ± 2.48	68.26 ^b^ ± 1.55	67.62 ^b^ ± 2.21	63.82 ^a^ ± 2.57	63.45 ^a^ ± 1.13	62.24 ^a^ ± 2.36	63.00 ^a^ ± 2.91	62.64 ^a^ ± 1.69
a*(−)	10.03 ^bc^ ± 1.28	8.31^a^ ± 0.88	8.22 ^a^ ± 0.83	10.48 ^c^ ± 0.76	8.23 ^a^ ± 0.88	10.31 ^c^ ± 0.91	9.35 ^abc^ ± 0.80	8.86 ^ab^ ± 0.79
b*(−)	8.21 ^a^ ± 0.60	8.63 ^ab^ ± 0.70	8.52 ^ab^ ± 0.46	8.46 ^ab^ ± 0.66	9.19 ^bc^ ± 0.52	9.69 ^cd^ ± 0.75	10.32 ^d^ ± 0.50	9.29 ^bc^ ± 0.69
Hardness (N)	79.84 ^c^ ± 9.84	69.26 ^abc^ ± 3.29	60.22 ^a^ ± 6.29	70.86 ^abc^ ± 2.85	75.27 ^b^ ± 6.42	74.83 ^b^ ± 2.70	71.47 ^abc^ ± 2.21	66.38 ^ab^ ± 3.10
Springiness [−]	0.37 ^bc^ ± 0.02	0.30 ^ab^ ± 0.06	0.48 ^d^ ± 0.02	0.34 ^b^ ± 0.03	0.35 ^b^ ± 0.05	0.45 ^d^ ± 0.02	0.44 ^cd^ ± 0.01	0.24 ^a^ ± 0.03
Cohesiveness [−]	0.34 ^abc^ ± 0.02	0.29 ^a^ ± 0.02	0.44 ^d^ ± 0.01	0.40 ^cd^ ± 0.01	0.33 ^ab^ ± 0.03	0.42 ^d^ ± 0.02	0.37 ^bcd^ ± 0.02	0.33 ^ab^ ± 0.06
Gumminess [−]	27.11 ^bc^ ± 3.98	20.14^a^ ± 1.86	26.42 ^abc^ ± 3.29	28.32 ^bc^ ± 1.35	24.82 ^a^ ± 3.91	31.60 ^c^ ± 2.29	26.37 ^abc^ ± 2.19	21.79 ^ab^ ± 3.60
Chewiness [N]	10.04 ^cd^ ± 1.40	6.07 ^ab^ ± 1.55	12.59 ^de^ ± 1.69	9.70 ^c^ ± 1.06	8.64 ^bc^ ± 0.68	14.29 ^e^ ± 0.48	11.48 ^cde^ ± 0.63	5.32 ^a^ ± 1.60

CON—control (water + salt); BL—bay leaf; BP—black pepper; TU—turmeric; TA—tamarind paste; JP—jalapeno pepper; MX—all spice mix; C—commercial marinade. ^a–e^—the mean values marked with various lowercase letters correspond to significant differences within rows (*p* < 0.05).

**Table 7:** The effect of marinade on PAH formation (mean ± standard deviation) in grilled pork.

PAH Compound	Abbreviation	PAH Concentration [mg/kg]							
		CON	BL	BP	TU	TA	JP	MX	C
Fluorene	F	0.37 ± 0.03 ^B^	0.33 ± 0.01 ^B^	1.39 ± 0.05 ^C^	0.00 ± 0.00 ^A^	0.00 ± 0.00 ^A^	0.00 ± 0.00 ^A^	0.00 ± 0.00 ^A^	1.88 ± 0.04 ^D^
Anthracene	ANT	0.50 ± 0.07 ^A^	0.35 ± 0.01 ^A^	5.14 ± 0.17 ^D^	0.49 ± 0.01 ^A^	0.36 ± 0.03 ^A^	0.54 ± 0.01 ^A^	0.35 ± 0.04 ^A^	3.12 ± 0.10 ^B^
Fluoranthene	FL	2.17 ± 0.09 ^D^	5.93 ± 0.37 ^F^	1.77 ± 0.12 ^CD^	0.88 ± 0.02 ^B^	1.62 ± 0.09 ^C^	0.28 ± 0.02 ^A^	1.47 ± 0.03 ^C^	3.18 ± 0.05 ^E^
Benzo[b]fluorene	BbF	29.57 ± 0.45 ^G^	16.14 ± 0.43 ^D^	21.54 ± 0.64 ^EF^	8.09 ± 0.37 ^B^	11.72 ± 0.46 ^C^	2.90 ± 0.11 ^A^	20.92 ± 0.36 ^E^	22.39 ± 0.81 ^F^
Benz[a]anthracene	BaA	3.18 ± 0.17 ^E^	1.62 ± 0.38 ^C^	2.22 ± 0.08 ^D^	0.37 ± 0.02 ^B^	0.00 ± 0.00 ^A^	0.20 ± 0.01 ^AB^	0.37 ± 0.03 ^B^	1.44 ± 0.06 ^C^
Chrysene	CHR	18.79 ± 1.05 ^F^	7.49 ± 0.06 ^D^	11.1 ± 0.18 ^E^	4.86 ± 0.23 ^C^	5.44 ± 0.21 ^C^	0.00 ± 0.00 ^A^	0.70 ± 0.02 ^B^	18.97 ± 0.29 ^F^
*Σlight PAHs*		54.58 ± 1.11 ^H^	31.86 ± 0.93 ^E^	43.16 ± 0.99 ^F^	14.69 ± 0.38 ^B^	19.13 ± 0.64 ^C^	3.92 ± 0.11 ^A^	23.81 ± 0.35 ^D^	50.98 ± 0.66 ^G^
Benzo[a]pyrene	BaP	1.70 ± 0.08 ^D^	1.50 ± 0.02 ^C^	1.46 ± 0.03 ^C^	1.30 ± 0.06 ^C^	1.11 ± 0.08 ^B^	0.00 ± 0.00 ^A^	1.44 ± 0.11 ^C^	1.66 ± 0.06 ^D^
Benzo[b]fluoranthene	BbFL	1.15 ± 0.11 ^F^	0.00 ± 0.00 ^A^	0.25 ± 0.05 ^C^	0.47 ± 0.04 ^DE^	0.13 ± 0.01 ^B^	0.41 ± 0.03 ^D^	0.56 ± 0.03 ^E^	1.47 ± 0.10 ^G^
Benzo[g.h.i]perylene	BghiP	0.79 ± 0.03 ^C^	0.43 ± 0.04 ^B^	0.43 ± 0.03 ^B^	0.00 ± 0.00 ^A^	0.43 ± 0.03 ^B^	0.43 ± 0.04 ^B^	0.45 ± 0.02 ^B^	1.70 ± 0.03 ^D^
Benzo[k]fluoranthene	BkF	1.47 ± 0.15 ^B^	0.00 ± 0.00 ^A^	0.14 ± 0.03 ^A^	0.00 ± 0.00 ^A^	0.00 ± 0.00 ^A^	0.00 ± 0.00 ^A^	0.00 ± 0.00 ^A^	3.04 ± 0.02 ^C^
Dibenz[a.h]anthracene	DBahA	38.14 ± 0.75 ^F^	7.87 ± 0.26 ^D^	0.51 ± 0.03 ^A^	14.97 ± 0.22 ^E^	0.63 ± 0.04 ^A^	0.00 ± 0.00 ^A^	6.64 ± 0.07 ^C^	2.71 ± 0.08 ^B^
Indeno[1.2.3-cd]pyrene	IP	0.00 ± 0.00	0.00 ± 0.00	0.00 ± 0.00	0.00 ± 0.00	0.00 ± 0.00	0.00 ± 0.00	0.00 ± 0.00	0.00 ± 0.00
*Σheavy PAHs*		43.90 ± 0.95 ^E^	9.80 ± 0.24 ^CD^	2.79 ± 0.03 ^B^	16.74 ± 0.27 ^D^	2.30 ± 0.11 ^B^	0.84 ± 0.06 ^A^	9.09 ± 0.04 ^C^	10.58 ± 0.04 ^D^
Σ4 PAHs		24.82 ± 0.91 ^G^	10.61 ± 0.36 ^D^	15.03 ± 0.20 ^E^	7.00 ± 0.30 ^C^	6.68 ± 0.15 ^C^	0.61 ± 0.02 ^A^	3.07 ± 0.09 ^B^	23.54 ± 0.30 ^F^
Σ12 PAHs		98.48 ± 0.81 ^G^	41.66 ± 1.15 ^D^	45.95 ± 1.02 ^E^	31.43 ± 0.60 ^C^	21.43 ± 0.54 ^B^	4.76 ± 0.08 ^A^	32.90 ± 0.37 ^C^	61.56 ± 0.66 ^F^

PAH—polycyclic aromatic hydrocarbons. CON—control (water + salt); BL—bay leaf; BP—black pepper; TU—turmeric; TA—tamarind paste; JP—jalapeno pepper; MX—all spice mix; C—commercial marinade. ^A–H^—the mean values marked with various capital letters correspond to significant differences within rows (*p* < 0.05).

**Table 8:** Correlation coefficient (p) between the level of PAHs (µg/kg) and DPPH and TPC.

	12 PAHs	ANT	BaA	BaP	BbFL	BbF	BghiP	BkF	CHR	DBahA	FL	F	Light PAHs	Heavy PAHs
DPPH	−0.779	0.101	−0.660	−0.251	−0.620	−0.503	−0.264	−0.465	−0.643	−0.879	0.131	−0.047	−0.524	−0.903
TPC	−0.576	−0.157	−0.624	−0.341	−0.140	−0.240	−0.013	−0.233	−0.639	−0.574	−0.203	−0.199	−0.458	−0.576

CON—control (water + salt); BL—bay leaf; BP—black pepper; TU—turmeric; TA—tamarind paste; JP—jalapeno pepper; MX—all spice mix; C—commercial marinade.
